# Single-cell RNA sequencing identifies distinct transcriptomic signatures between PMA/ionomycin- and αCD3/αCD28-activated primary human T cells

**DOI:** 10.5808/gi.23009

**Published:** 2023-06-30

**Authors:** Jung Ho Lee, Brian H Lee, Soyoung Jeong, Christine Suh-Yun Joh, Hyo Jeong Nam, Hyun Seung Choi, Henry Sserwadda, Ji Won Oh, Chung-Gyu Park, Seon-Pil Jin, Hyun Je Kim

**Affiliations:** 1 Department of Biomedical Sciences, Seoul National University Graduate School, Seoul 03080, Korea; 2 Department of Anatomy, Yonsei University College of Medicine, Seoul 03722, Korea; 3 Department of Microbiology and Immunology, Seoul National University College of Medicine, Seoul 03080, Korea; 4 Transplantation Research Institute, Seoul National University College of Medicine, Seoul 03080, Korea; 5 Department of Dermatology, Seoul National University Hospital, Seoul 03080, Korea; 6 Department of Dermatology, Seoul National University College of Medicine, Seoul 03080, Korea; 7 Medical Research Center, Institute of Human-Environmental Interface Biology, Seoul National University College of Medicine, Seoul 03080, Korea; 8 Genomic Medicine Institute, Seoul National University College of Medicine, Seoul 03080, Korea; 9 Seoul National University Hospital, Seoul 03080, Korea

**Keywords:** scRNA-seq, T cell, T cell activation, transcriptome

## Abstract

Immunologists have activated T cells *in vitro* using various stimulation methods, including phorbol myristate acetate (PMA)/ionomycin and αCD3/αCD28 agonistic antibodies. PMA stimulates protein kinase C, activating nuclear factor-κB, and ionomycin increases intracellular calcium levels, resulting in activation of nuclear factor of activated T cell. In contrast, αCD3/αCD28 agonistic antibodies activate T cells through ZAP-70, which phosphorylates linker for activation of T cell and SH2-domain-containing leukocyte protein of 76 kD. However, despite the use of these two different *in vitro* T cell activation methods for decades, the differential effects of chemical-based and antibody-based activation of primary human T cells have not yet been comprehensively described. Using single-cell RNA sequencing (scRNA-seq) technologies to analyze gene expression unbiasedly at the single-cell level, we compared the transcriptomic profiles of the non-physiological and physiological activation methods on human peripheral blood mononuclear cell–derived T cells from four independent donors. Remarkable transcriptomic differences in the expression of cytokines and their respective receptors were identified. We also identified activated CD4 T cell subsets (CD55^+^) enriched specifically by PMA/ionomycin activation. We believe this activated human T cell transcriptome atlas derived from two different activation methods will enhance our understanding, highlight the optimal use of these two *in vitro* T cell activation assays, and be applied as a reference standard when analyzing activated specific disease-originated T cells through scRNA-seq.

## Introduction

The human immune system has evolved to survive against a variety of infections. T cells, which are among the central players in the adaptive immune system, not only recognize and eradicate infected cells, but also interact with other immune cells through cytokines. Hence, the production of cytokines from activated T cells is an essential process for protecting the body, mediating inflammation, and regulating other types of immune cells. T cells need several simultaneous signals to be fully activated, including T cell receptor (TCR), costimulatory, and cytokine signals. To study the detailed activation steps and role of activated T cells in specific environments, immunologists have used two different methods to activate T cells: αCD3/αCD28 agonistic antibodies and phorbol 12-myristate 13-acetate (PMA)/ionomycin.

Anti-CD3 is an agonistic antibody that can physiologically stimulate the TCR, thereby activating ZAP-70, which is the initiator of T cell downstream signaling [1]. ZAP-70 delivers downstream signals through phosphorylation of its primary targets: linker for activation of T cell (LAT) and SH2-domain-containing leukocyte protein of 76 kD (SLP-76) [2,3]. After the activation of LAT and SLP-76, several signaling molecules are recruited, including phospholipase C-γ (PLC-γ) and AKT, which play a key role in cellular metabolism. In combination, anti-CD28 maximizes PLC-γ activation, generating two second messengers—diacylglycerol (DAG) and IP3, from PIP2—via the local production of PIP3 [4]. In contrast, PMA induces Ras and protein kinase C to activate nuclear factor-κB in a SOS- and CARMA1-dependent manner [5]. Ionomycin triggers a cytosolic influx of calcium ions, effectively simulating the role of PLC-γ. However, differences in the resulting T cell gene expression profiles between the two activation methods using single-cell transcriptomic techniques have not yet been explored.

In this study, we used single-cell RNA sequencing to compare resting, αCD3/αCD28 agonistic antibody-activated, and PMA/ionomycin-activated T cells isolated from peripheral blood mononuclear cells (PBMCs) from four healthy individuals. By analyzing a total of 35,736 resting, PMA/ionomycin-activated, and αCD3/αCD28 agonistic antibody-activated T cells, we aimed to establish a standard reference that would aid researchers in determining which activation method to apply and could be used to analyze specific disease-derived T cells after activation. 

## Methods

### Isolation of PBMCs

The collection of healthy individuals’ blood was approved by the Seoul National University Hospital Institutional Review Board (SNUH IRB No. C-2205-189-1327). From each donor, 16 mL of whole blood was collected in heparin CPT tubes (cat No. 362753, BD, Franklin Lakes, NJ, USA). The collected blood was immediately processed for PBMC isolation. CPT tubes were centrifuged for 20 min at 1,800 ×g with minimum acceleration and deceleration, and the interphase was collected in complete medium (RPMI containing 10% fetal bovine serum [FBS]). After washing with complete medium twice, the PBMCs were frozen with Cell Banker I (cat No. 11888, AMSBIO, Abingdon, UK) and stored in liquid nitrogen until batch processing.

### T cell enrichment and stimulation

T cells were enriched using the EasySep human T cell enrichment kit (cat No. 19051, STEMCELL Technologies, Vancouver, Canada). After T cell enrichment, the cells were suspended in complete medium (RPMI 1640, 50%, Gibco, Thermo Fisher Scientific, Waltham, MA, USA) containing NEAA, HEPES, L-GlutaMax, 2-mercaptoethanol, FBS, and antibiotics. Brefeldin A was added to all groups in order to lock the produced transcripts and proteins inside the cells. Interleukin (IL)-2 was added to all groups to ensure T cell survival during the incubation period. All donor samples were divided into three activation groups: group 1, resting control; group 2, 50 ng/mL PMA (cat No. P8139, 1 mg, Sigma Aldrich, St. Louis, MO, USA) and 1.34 µM ionomycin (cat No. I0634, 1 mg, Sigma) for chemical stimulation and group 3, Dynabeads human T-activator CD3/CD28 (cat No. 11131D, Gibco, Thermo Fisher Scientific) added at a 1:1 ratio (beads: cells) for physiological activation. Each group was incubated for 4 h at 37.5℃ in a 5% CO_2_ incubator. After 4 h of incubation, the cells were harvested and used in further experiments.

### Purity and protein validation through flow cytometry

An aliquot of the T cell-enriched samples from each donor was stained with anti-human CD3 (cat No. 300426, clone UCHT1, BioLegend, San Diego, CA, USA) for 30 minutes at room temperature to validate the isolation of T cells. The aliquots were also stained with anti-human CD3 (cat No. 300426, clone UCHT1, BioLegend), anti-human CD8 (cat No. 560662, clone RPA-T8, BD Biosciences, Franklin Lakes, NJ, USA), and anti-human CD45RO (cat No. 562299, clone UCHL1, BD Biosciences). The cells were then treated with fixation/permeabilization concentrate (cat No. 00-5123-43, Invitrogen, Waltham, MA, USA) to fix and permeabilize cells for 1 h at 4°C, and intracellular staining was conducted according to the manufacturer’s recommendations. The cells were stained with anti-human interferon γ (IFN-γ) (cat No. 56-7319-41, clone 4S.B3, Invitrogen), anti-human tumor necrosis factor α (TNF-α) (cat No. 25-7349-41, clone MAb11, Invitrogen), and anti-human IL-2 (cat No. 500307, clone MQ1-17H12, BioLegend). Sample data were acquired using the LSR Fortessa X20 and were analyzed using FlowJo software.

### Cell multiplexing

For each experimental condition, samples from four donors were multiplexed. Immediately following T cell activation, all 12 samples (4 donors × 3 conditions each) were multiplexed in Cell Multiplexing Oligo (CMO) for 5 min at room temperature using the 3′ Cellplex kit set A (PN 1000261) according to the manufacturer’s recommendation to reduce batch effects. The labeled cells were washed several times to avoid multi-labeling after pooling of cells. The labeled cells were washed by centrifugation at 400 ×g at 4°C following the 10× Genomics protocol with RPMI containing 10% FBS.

### Single-cell RNA library construction and sequencing

Gene expression and CMO libraries were constructed following the 10× Genomics guidelines. The 4150 TapeStation system (Agilent, Santa Clara, CA, USA) was used for quality control of the cDNA and cell multiplexing libraries. Sequencing was done by NovaSeq 6000 (Illumina, San Diego, CA, USA) at a depth of 50,000 and 10,000 reads per cell for gene expression and CMO libraries, respectively.

### Data analysis

Demultiplexing and alignment to the human genome were performed using the Cell Ranger software (v6.1.2). The Seurat package (v4.2) was used for pipeline analysis of the aligned dataset. All data were integrated using Harmony (v1.0) to minimize batch effects. Doublets were excluded using Doublet Finder (v2), and cells with over 3,300 or fewer than 200 features of transcripts were excluded. Furthermore, cells showing a percentage of mitochondrial gene expression exceeding 7.5% were excluded. Non-T cells were excluded based on the absence of *CD3E*, *CD3D*, *CD247*, and *CD3G* expression. Normalization was applied using the log normalization method. We also applied the Louvain algorithm for clustering, and differentially expressed genes were identified using the Wilcoxon rank-sum test. The Monocle3 (v1.3.1) package was used to order the cells according to the pseudotime via trajectory analysis with 0.9 resolution, 10 principal components, and naïve T cells selected as root nodes.

### Data available

The primary human T cell scRNA-seq data used in this study are available with links to BioProject accession number PRJNA948720 in the NCBI BioProject database (https://www.ncbi.nlm.nih.gov/bioproject/).

###  

## Results

### scRNA-seq data generation from multiplexed human activated T cells

**Fig. 1.**
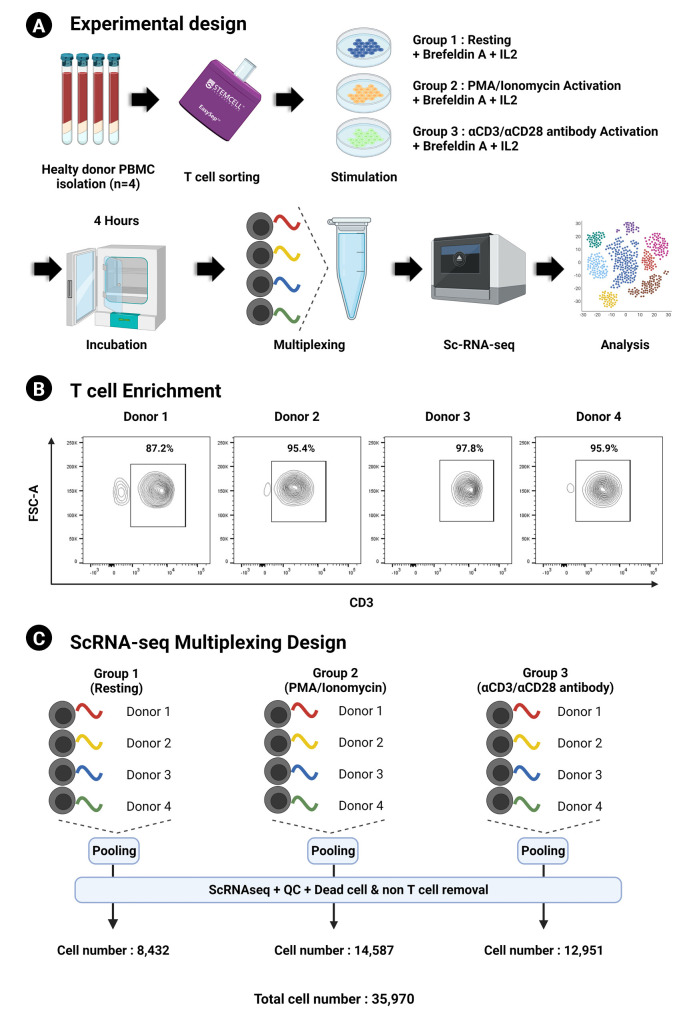
Workflow of the experiment. (A) Overview of the experimental design. (B) CD^3+^ sorting purity validation using flow cytometry. (C) Design of the scRNA-seq multiplexing and total recovered cell number. All figures were created with BioRender.com. IL-2, interleukin 2; PBMC, peripheral blood mononuclear cell; scRNA-seq, single-cell RNA sequencing.

The experimental design is summarized in Fig. 1A. The results of sorting CD3^+^ T cells from PBMCs were validated by flow cytometry with an obtained sorting purity of over 85% T cells from all donors (Fig. 1B). Following activation in each experimental condition (control, PMA/ionomycin, and αCD3/αCD28), we obtained a total of 35,970 cells (8,432 cells in the resting control group, 14,587 cells in the PMA/ionomycin group, and 12,951 cells in the αCD3/αCD28 agonistic antibody group) (Fig. 1C).

### Frequency of annotated cell type across donors and experimental conditions

**Fig. 2.**
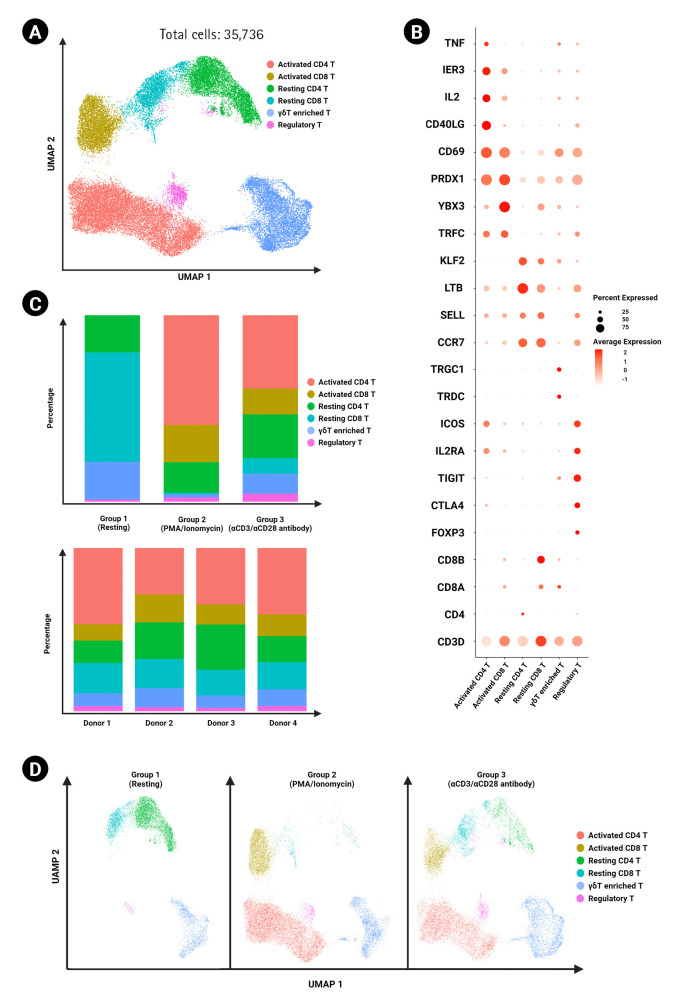
Annotation of total T cells and comparison of frequencies across experimental conditions. (A) UMAP of 35,736 T cells clustered by cell type and activation markers. Clusters are color-coded. (B) Dot plot showing expression levels of canonical markers in each cluster. The percent expression of CD^4+^ and CD^8+^ T cell genes is represented based on the dot size, and the average expression is represented by color. (C) The proportions of CD^4+^ and CD^8+^ T cell clusters were grouped by experimental conditions (top) and donors (bottom). (D) UMAP of CD^4+^ and CD^8+^ T cells divided by different experimental conditions.

T cells across all experimental conditions and donors were integrated using the top 1,000 highly variable genes (Fig. 2A). Unbiased clustering was performed using the Louvain clustering method, and T cell clusters were annotated using cell type- and activation type-specific gene markers (Fig. 2B). The frequencies of annotated cell types by each experimental condition and donor are shown in Fig. 2C. No significant differences were found in cell type frequency across donors. Activated CD4 T cells and activated CD8 T cells were found at higher frequencies in the PMA/ionomycin-stimulated activation group than in the agonistic antibody-stimulated group (Fig. 2D).

### Cytokine and cytokine receptor expression on activated CD4 T cells

**Fig. 3.**
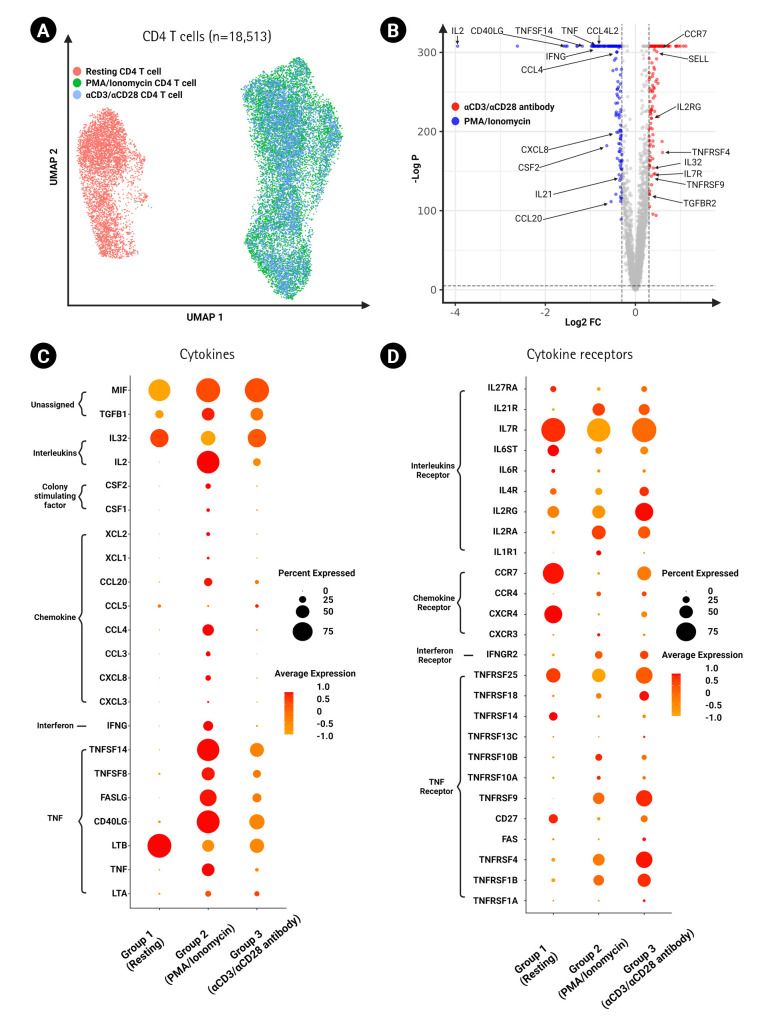
Expression of cytokines and cytokine receptors on CD^4+^ T cells. (A) UMAP of CD^4+^ T cells (18,513 cells) clustered by different experimental conditions. (B) Volcano plot showing differentially expressed genes between αCD3/αCD28 antibody-activated CD^4+^ T cells and phorbol myristate acetate (PMA)/ionomycin-activated CD^4+^ T cells. Dot plot showing the expression of cytokine genes (C) and cytokine receptor genes (D) in activated CD^4+^ T cells between the two groups of distinct activation methods.

To further interrogate the various CD4 T cell activation states induced by the different stimulation methods, we grouped the resting and activated CD4 T cells (total of 18,513 cells) and applied unsupervised sub-clustering (Fig. 3A). We found broad differences between the stimulation methods regarding the gene expression of cytokines and their respective receptors (Fig. 3B). Cytokine expression, including *TGFB1*, *IL2*, *CSF1*, *CSF2*, *XCL1*, *XCL2*, *CCL20*, *CCL4*, *CCL3*, *CXCL8*, *CXCL3*, *IFNG*, *TNFSF14*, *TNFSF8*, *FASLG*, *CD40LG*, and *TNF*, was higher in the PMA/ionomycin group than in the αCD3/αCD28 agonistic antibody-treated group in terms of both percentages of expression and average expression per cell. The percentages of expression of *MIF* and *LTA* were similar between the two activated groups. However, the expression levels and percentages of expression of *IL32* and *CCL5* were higher in the αCD3/αCD28 agonistic antibody-treated group (Fig. 3C). For cytokine receptor expression, the TNF superfamily, including *TNFRSF1A*, *TNFRSF1B*, *TNFRSF4*, *FAS*, *CD27*, *TNFRSF9*, *TNFRSF18*, and *TNFRSF25*, showed overall elevated expression in the αCD3/αCD28 agonistic antibody-treated group. Furthermore, receptors including *CXCR4*, *CCR7*, *IL2RG*, *IL4R*, *IL6ST*, *IL7R*, and *IL27RA* showed higher expression in the αCD3/αCD28 agonistic antibody-treated group (Fig. 3D). Conversely, *IL2RA*, *IL1R1*, and *CXCR3* were higher in the PMA/ionomycin group. Among these, IFN-γ, TNF-α, and IL-2 were validated at the protein level using flow cytometry, and the findings were found to be consistent with the transcriptomic levels (Supplementary Fig. 1A).

### Cytokine and cytokine receptor expression on activated CD8 T cells

**Fig. 4.**
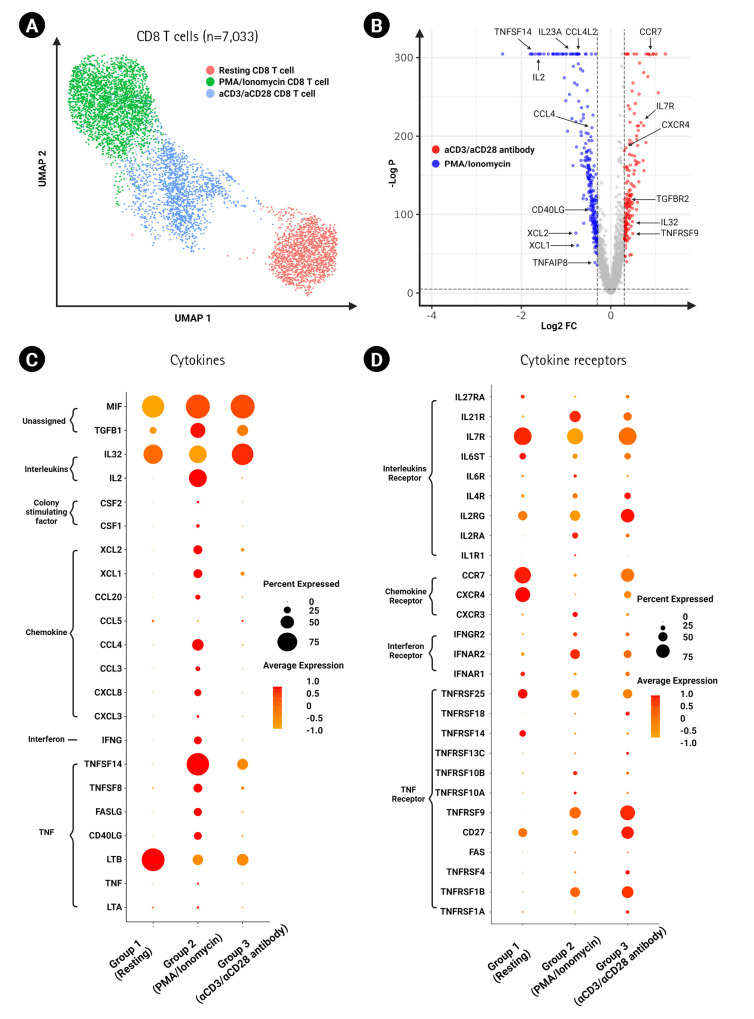
Expression of cytokines and cytokine receptors on CD^8+^ T cells. (A) UMAP of CD^8+^ T cells (7,033 cells) clustered by different experimental conditions. (B) Volcano plot showing differentially expressed genes between αCD3/αCD28 antibody-activated CD^8+^ T cells and phorbol myristate acetate (PMA)/ionomycin-activated CD^8+^ T cells. Dot plot showing the expression of cytokine genes (C) and cytokine receptor genes (D) in activated CD^8+^ T cells between the two groups of distinct activation methods.

We applied the same sub-clustering strategy as described above for sub-clustering CD8 T cells (Fig. 4A). We grouped and then sub-clustered a total of 7,033 resting and activated CD8 T cells, again finding unique expression patterns in terms of cytokines and their respective receptor expression (Fig. 4B). We found similarities in cytokine expression between activated CD8 T cells and CD4 T cells, but the expression of *TNF* was distinctly reduced in activated CD8 T cells (Fig. 4C). In addition, the expression patterns of cytokine receptors were similar as those in CD4 T cells, but *IL2RA* showed lower levels of expression, and *IFNAR1* and *IFNAR2* were distinctive in CD8 T cells (Fig. 4D). *IL6R* expression was elevated in the cells that received PMA/ionomycin stimulation. The protein levels of IFN-γ, TNF-α, and IL-2 were also validated in CD8 T cells by flow cytometry and were consistent with the transcriptome levels (Supplementary Fig. 1B).

### Enrichment of CD55+ activated CD4 T cells by PMA stimulation

**Fig. 5.**
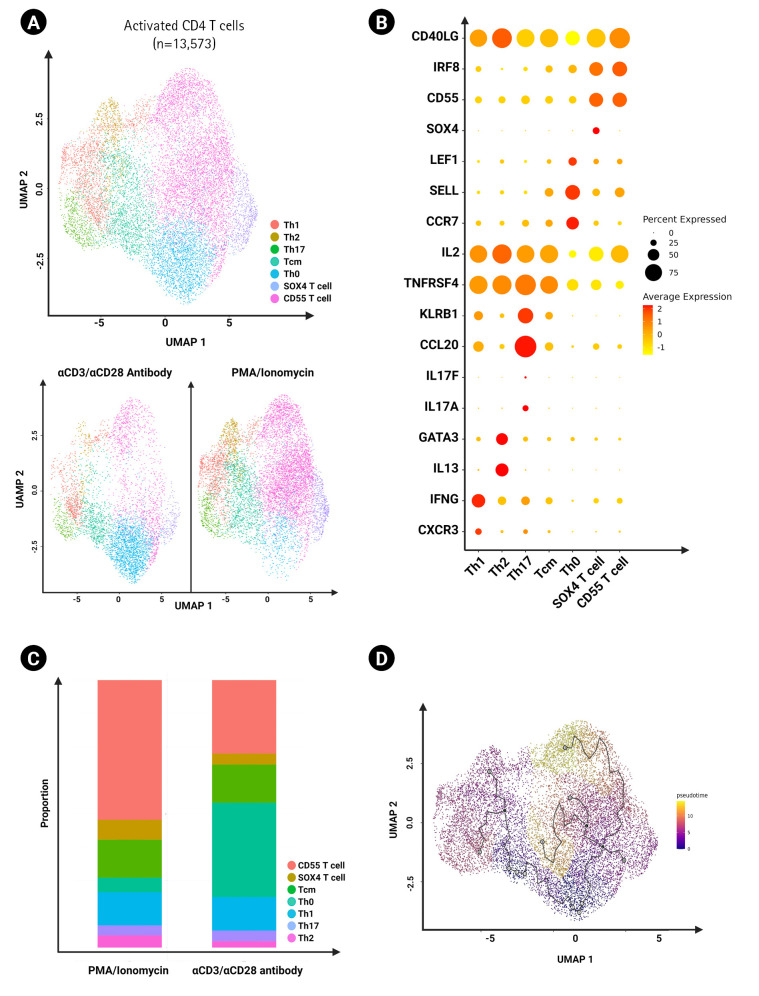
Characterization of activated CD^4+^ T cells. (A) UMAP of activated CD^4+^ T cells (13,573 cells) clustered by different CD^4+^ subsets in different experimental conditions. (B) Dot plot showing the expression of cytokine genes in CD^4+^ T cell subsets between the two groups of distinct activation methods. (C) Bar graph showing frequency of each cluster of activated CD^4+^ T cells using the two distinct activation methods. (D) UMAP of trajectory analysis on activated CD^4+^ T cells reported by Monocle 3.

We grouped all the activated CD4 T cells across all stimulated groups and sub-clustered them for further analysis (a total of 13,573 cells). We annotated the subsets based on differential gene marker expression and found Th1, Th2, Th17, central memory helper T cell, activated naïve helper T cell, SOX4 T cell and CD55^+^ T cells (Fig. 5B). We found that CD55^+^ T cells were enriched specifically by PMA/ionomycin activation (Fig. 5C). Moreover, the Monocle 3 package was used to order the cells according to pseudotime via trajectory analysis (Fig. 5D). Using Monocle 3 for pseudotime trajectory analysis, we found that CD55^+^ T cells and SOX4 T cells could potentially be differentiated from naïve T cells instead of central memory T cells.

## Discussion

Naïve T cells require two different extracellular signals in order to become activated. Each T cell has its antigen specificity, and if this receptor interacts with the antigen presented by the MHC-antigen peptide complex on the antigen-presenting cell (APC), an initial signal is generated. Signaling is transmitted into the cells via CD3 [6,7]. A secondary signal is also required from costimulatory molecules on the APC and the corresponding ligand on the T cell surface [8]. Therefore, stimulating T cells with αCD3/αCD28 antibodies closely resembles the natural downstream signal of the TCR, resulting in a more physiological method for activating T cells. However, αCD3/αCD28 agonistic antibodies may indirectly activate other classes of lymphoid-lineage cells, such as T cells, B cells, and natural killer cells, upon activation [9]. In addition, the heterogeneity of CD3 expression among T cell subsets may lead to varying degrees of T cell activation [10,11], and these factors should be considered for precise data interpretation.

PMA, a DAG analog, exerts its effect on protein kinase C by crossing the cell membrane. Ionomycin increases intracellular calcium ions and activates calcineurin. The shared signals of protein kinase C and calcineurin promote the activation of T cells [12]. PMA/ionomycin is widely employed to stimulate immune cells since it is relatively inexpensive and simple to optimize the conditions [13]. However, when applying this method for T cell activation, it should be noted that PMA/ionomycin stimulates cells through non-specific mechanisms and can be toxic to cells due to overstimulation, leading to activation-induced cell death.

As predicted, the percentage of activated T cells was higher in the stimulated groups than in the resting group. We also found that the PMA/ionomycin-mediated activation group had a higher frequency of activated CD4 T cells and proportionally fewer resting CD4 T cells than the αCD3/αCD28 agonistic antibody group (Fig. 2C). Activation markers, including *CD69*, *IL2RA*, *CD40LG*, *ICOS*, *CTLA4*, and *PDCD1*, were present at elevated levels on PMA/ionomycin-treated T cells when compared to the αCD3/αCD28 agonistic antibody-treated T cells (Supplementary Fig. 2). Taken together, these results indicate that PMA/ionomycin has a greater T cell-stimulating potential than the αCD3/αCD28 agonistic antibodies under the same duration of incubation. However, not all genes related to T cell activation showed higher expression in the PMA/ionomycin-stimulated group in analyses of cytokines and their receptors.

Cytokines are critical to T cell function in terms of maturation, growth, and response to stimuli. Antigen stimulation induces T cell activation and the production of numerous cytokines in peripheral T cells (Figs. 3 and 4) [14]. We identified several cytokines among the differentially expressed genes in activated T cells according to the activation method (Figs. 3C and 4C). Generally, cytokine expression was higher in the PMA/ionomycin-treated group than in the αCD3/αCD28 agonistic antibody-treated group, including the TNF family. Since the members of the TNF family (*TNFSF14*, *TNFSF8*, *FASLG*, and *TNF*) are related to apoptosis, this result is aligned with a previous report stating that PMA/ionomycin stimulation results in activation-induced cell death via Fas/Fas ligand up-regulation [15]. A study using CD4 T cells from intestinal biopsies also showed that PMA/ionomycin induced larger amounts of IFN-γ and IL-17, while IL-10 production was predominant following αCD3/αCD28 stimulation [13].

Cytokines deliver signals upon binding to their receptors. A greater number of receptors on a cell indicates that it is more sensitive to a cytokine. Here, we found that, in general, the expression of cytokine receptors in the αCD3/αCD28 agonistic antibody-treated group was greater than that in the PMA/ionomycin-treated group. Only the genes for a few receptors, such as *IL2RA*, *IL1R1*, *TNFRSF10B*, and *TNFRSF10A*, were elevated in the PMA/ionomycin-treated group.

As for both cytokines and their receptors, activated CD8 T cells showed similar findings to activated CD4 T cells upon two different stimulation methods, suggesting a general stimulatory effect on T cell subsets (Fig. 4C and 4D).

When further investigating the activated CD4 T cell subsets, the proportions of Th1, Th2, and Th17 were not remarkably different despite the higher potential of PMA/ionomycin to induce cytokines (Fig. 5C). However, we found an increase in activated CD4 T cells expressing CD55 in response to PMA/ionomycin, and trajectory analysis showed that their potential source was naïve CD4 T cells (Fig. 5C and D). Further studies are needed to elucidate the role of this population.

The main limitation of this study is that we utilized a single fixed experimental condition for the two distinct stimulation methods. The results may vary depending on the concentration and length of incubation. Nonetheless, we anticipate that our research will aid in determining the most appropriate stimulation methods for T cell research based on the objectives of the study.

## Supplementary Materials

Supplementary Fig. 1. Validation of scRNA-seq results at the protein level using flow cytometry. (A,B) Expression of IFN-γ, TNF-α, and IL-2 on (A) CD4 T cells (gated by CD4+CD8-) and (B) CD8 T cells (gated by CD4-CD8+). IFN-γ, interferon γ; IL-2, interleukin 2; scRNA-seq, single-cell RNA sequencing; TNF-α, tumor necrosis factor α. Statistical analysis was conducted with one-way ANOVA Dunnett’s multiple comparisons test. An asterisk (*) indicates significant differences compared to the resting group. *p < 0.05, **p < 0.01, ***p < 0.001, ****p < 0.0001.

Supplementary Fig. 2. Dot plot showing activation marker expression on T cells across experimental conditions.
