# Supplementary material for: Single-cell RNA sequencing identifies distinct transcriptomic signatures between PMA/ionomycin- and αCD3/αCD28-activated primary human T cells
Source: Genomics Inform. 2023 Jun 30;21(2):e18. doi: 10.5808/gi.23009 (PMC10326540; doi:10.5808/gi.23009)

Supplementary Fig. 1. Validation of scRNA-seq results at the protein level using flow cytometry. (A,B) Expression of IFN-γ, TNF-α, and IL-2 on (A) CD4 T cells (gated by CD4+CD8-) and (B) CD8 T cells (gated by CD4-CD8+). IFN-γ, interferon γ; IL-2, interleukin 2; scRNA-seq, single-cell RNA sequencing; TNF-α, tumor necrosis factor α. Statistical analysis was conducted with one-way ANOVA Dunnett's multiple comparisons test. An asterisk (*) indicates significant differences compared to the resting group. $^{*}p < 0.05$, $^{**}p < 0.01$, $^{***}p < 0.001$, $^{****}p < 0.0001$.

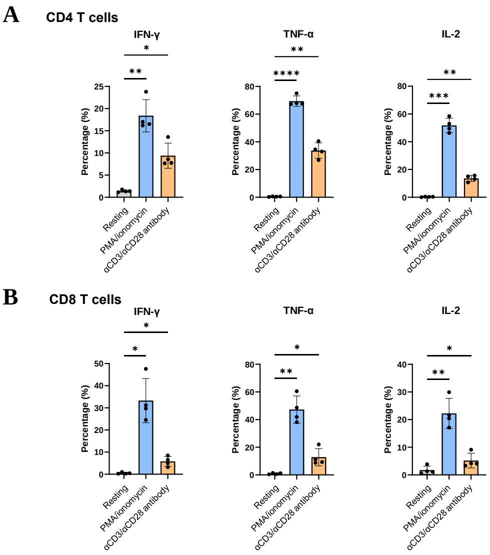

Supplement: Supplementary Fig. 1. — Validation of scRNA-seq results at the protein level using flow cytometry. (A,B) Expression of IFN-γ, TNF-α, and IL-2 on (A) CD4 T cells (gated by CD4+CD8-) and (B) CD8 T cells (gated by CD4-CD8+). IFN-γ, interferon γ; IL-2, interleukin 2; scRNA-seq, single-cell RNA sequencing; TNF-α, tumor necrosis factor α. Statistical analysis was conducted with one-way ANOVA Dunnett’s multiple comparisons test. An asterisk (*) indicates significant differences compared to the resting group. *p < 0.05, **p < 0.01, ***p < 0.001, ****p < 0.0001. [file gi-23009-Supplementary-Figure-1.pdf]
