# Supplementary material for: Single-cell RNA sequencing identifies distinct transcriptomic signatures between PMA/ionomycin- and αCD3/αCD28-activated primary human T cells
Source: Genomics Inform. 2023 Jun 30;21(2):e18. doi: 10.5808/gi.23009 (PMC10326540; doi:10.5808/gi.23009)

Supplementary Fig. 2. Dot plot showing activation marker expression on T cells across experimental conditions.

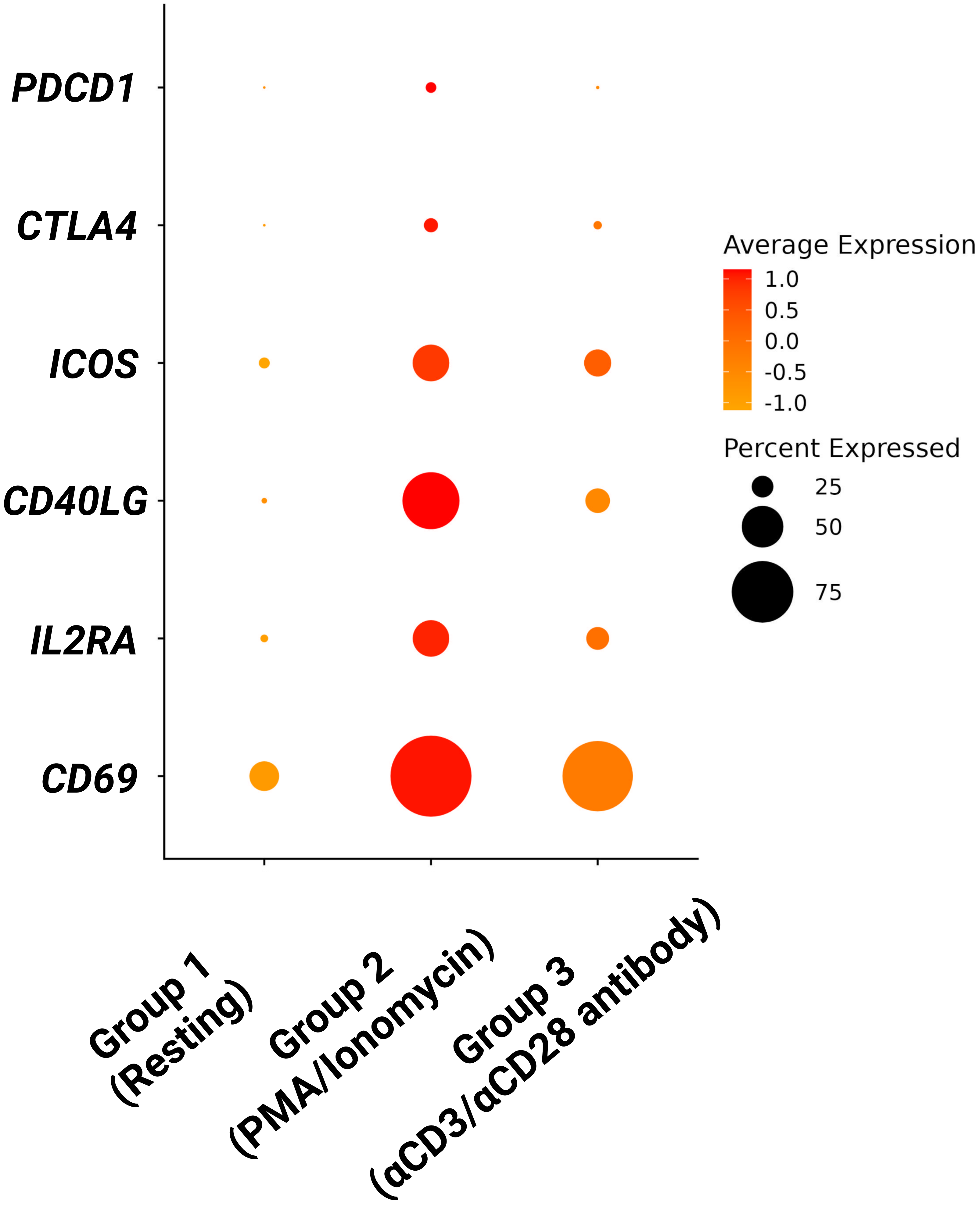

Supplement: Supplementary Fig. 2. — Dot plot showing activation marker expression on T cells across experimental conditions. [file gi-23009-Supplementary-Figure-2.pdf]
